# Changes in the acoustic activity of beaked whales and sperm whales recorded during a naval training exercise off eastern Canada

**DOI:** 10.1038/s41598-022-05930-4

**Published:** 2022-02-07

**Authors:** Joy E. Stanistreet, Wilfried A. M. Beslin, Katie Kowarski, S. Bruce Martin, Annabel Westell, Hilary B. Moors-Murphy

**Affiliations:** 1 Fisheries and Oceans Canada, Bedford Institute of Oceanography, 1 Challenger Drive, Dartmouth, NS Canada; 2 JASCO Applied Sciences, 32 Troop Avenue, Suite 202, Dartmouth, NS Canada

**Keywords:** Conservation biology, Marine biology

## Abstract

Experimental research has shown that beaked whales exhibit strong avoidance reactions to naval active sonars used during antisubmarine warfare training exercises, including cessation of echolocation and foraging activity. Behavioural responses to sonar have also been linked to strandings and mortality. Much of the research on the responses of beaked whales and other cetaceans to naval active sonar has occurred on or near U.S. naval training ranges, and the impacts of sonar in other regions remain poorly understood, particularly as these impacts, including mortality, are likely to go unobserved in offshore areas. In September 2016 the multinational naval exercise ‘CUTLASS FURY 2016’ (CF16) was conducted off eastern Canada. We used passive acoustic recordings collected in the region to quantify the occurrence and characteristics of sonar signals, measure ambient noise levels, and assess changes in the acoustic activity of beaked and sperm whales. The number of hours per day with echolocation clicks from Cuvier’s beaked whales and sperm whales were significantly reduced during CF16, compared to the pre-exercise period in 2016 (sperm whales) and to control data from 2015 (both species). Clicks from an unidentified Mesoplodont beaked whale species, sporadically detected prior to CF16, were absent during the exercise and for 7 days afterward. These results suggest that beaked and sperm whales ceased foraging in the vicinity of CF16 and likely avoided the affected area. Such disturbance may have energetic, health, and fitness consequences.

## Introduction

Toothed whales (Odontoceti) rely on specialized acoustic sensory systems for many life functions including foraging, communication, and avoiding predation. Anthropogenic noise introduced into the marine environment can disrupt the normal behaviours of these species and affect health, survival, and reproduction. The most extreme impacts of anthropogenic noise documented for odontocetes are dozens of fatal individual and mass strandings of beaked whales (family Ziphiidae) linked to the use of naval active sonars by the world’s militaries since the 1960s^1–4^. Concerns over the impacts of naval active sonar on beaked whales have prompted a substantial amount of research investigating the mechanistic links between sonar exposure and strandings^5,6^, the behavioural responses of individuals to sonar signals e.g.,^7–10^, and the energetic, fitness, and population consequences of sublethal behavioural disturbance^11–13^.

Beaked whales are deep-diving predators, routinely performing foraging dives to depths greater than 1000 m. Many beaked whale species are known to exhibit unique diving and vocal behaviour including silent descents and ascents and coordination among individuals when diving, potentially to reduce predation risk from killer whales (*Orcinus orca*)^14^. Strong avoidance responses to naval active sonar signals have been demonstrated in several different regions of the world by Blainville’s beaked whales (*Mesoplodon densirostris*)^7^, Cuvier’s beaked whales (*Ziphius cavirostris*)^8,15^, Baird’s beaked whales (*Berardius bairdii*)^16^, and northern bottlenose whales (*Hyperoodon ampullatus*)^9,10^, suggesting that these signals are widely perceived by beaked whales as a potential threat. The reactions observed during controlled experimental exposures to sonar signals typically included altered dive patterns, directed movement away from the sound source, and cessation of foraging activity. The severity of these responses varied across individuals, contexts, and studies. In nearly all cases, however, whales stopped producing echolocation clicks, indicating a switch to a non-foraging behavioural state, even as they performed longer and sometimes deeper dives^8–10^. Abrupt changes in diving behaviour in response to a perceived threat are thought to contribute to the formation of nitrogen gas bubbles and symptoms of decompression sickness which have been found in animals stranded in association with naval exercises employing active sonars^5,17,18^.

While the responses exhibited by beaked whales appear to be similar across species and genera, other deep-diving odontocetes have demonstrated a wider range of behavioural responses to sonar exposure. For example, long-finned pilot whales (*Globicephala melas*) rely on social defense rather than evasion of predators, and experimental exposure to simulated sonar signals has triggered responses such as increased aggregation and social cohesion^19^ with avoidance behaviour occurring only at high received sound levels^20^. Sperm whales (*Physeter macrocephalus*) have demonstrated varying responses to sonar exposure, including the interruption of foraging behaviour^21^. The behavioural responses of sperm whales have been studied experimentally^22,23^, but less extensively than beaked whales, and the potential impacts of full-scale military exercises on sperm whales are not known.

In September 2016, a multinational anti-submarine warfare training exercise named ‘CUTLASS FURY 2016’ (CF16) was conducted along the Scotian Shelf off eastern Canada, comprising the first event in a planned series of exercises hosted approximately biennially by Maritime Forces Atlantic (https://www.canada.ca/en/department-national-defence/news/2016/09/royal-canadian-navy-concludes-successful-anti-submarine-warfare-drills-john.html)^24^. Vessels, aircraft, and submarines from Canada, the United States, the United Kingdom, Spain, and France participated in this naval warfare simulation activity over a 12-day period. Military training activity at this scale does not commonly occur off the east coast of Canada, and cetaceans in this region are unlikely to regularly encounter extensive naval sonar activity in their acoustic environment. The Scotian Shelf and slope region are home to a diverse assemblage of cetacean species, including sperm whales and at least four species of beaked whales inhabiting deep waters along the continental shelf slope and beyond. Ongoing broad-scale passive acoustic monitoring efforts are providing insight into the seasonal occurrence and distributions of these species throughout the region, particularly within the Gully Marine Protected Area (MPA) located along the eastern edge of the Scotian Shelf^25,26^.

Here, we analyzed passive acoustic recordings collected off the Scotian Shelf during the CF16 exercise to assess changes in acoustic activity of beaked whales and sperm whales associated with the presence of naval active sonar signals. This study is observational and was developed opportunistically after we discovered sonar signals associated with the CF16 exercise in passive acoustic recordings collected along the western Scotian Shelf in 2016. We analyzed the surrounding 3-month recording period for the presence of beaked whale and sperm whale echolocation clicks, which are acoustically identifiable to the species level, and compared the results to recordings collected from the same location in 2015, when no military exercise occurred, to assess deviations from normal patterns of acoustic activity in each species. Additionally, we analyzed recordings collected during CF16 at two other sites, located within and near the Gully MPA, to illustrate both the geographic scale over which sonar signals were recorded during this exercise and the diversity of beaked whale species present across the region.

## Results

### Acoustic presence of sperm whales and beaked whales

**Figure 1:**
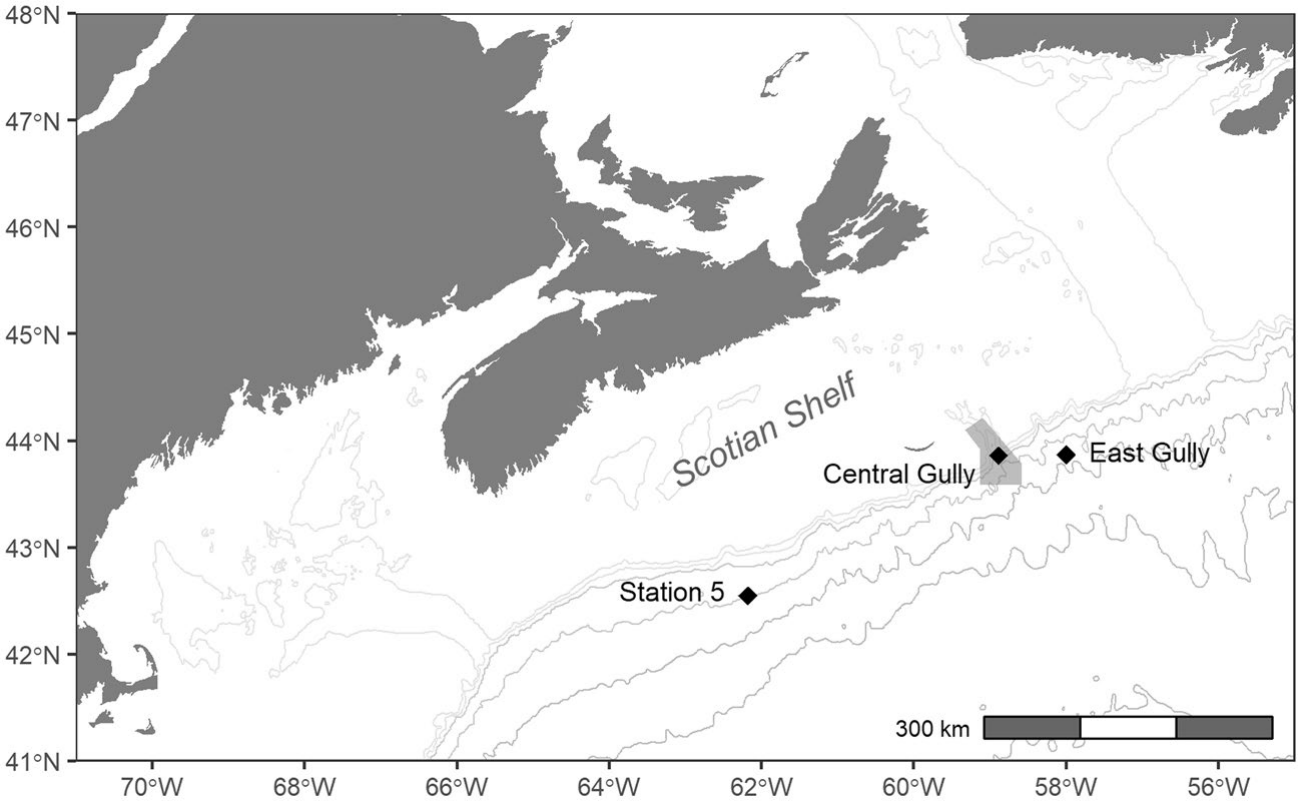
Map of recording sites located along the Scotian Shelf off eastern Canada. Shaded polygon around the Central Gully site delineates the Gully Marine Protected Area.

We analyzed acoustic data from a total of 243 recording days across the three recording sites (Fig. 1), and identified the hourly presence of echolocation clicks from beaked whales and sperm whales. Clicks from four beaked whale species were present in the recordings: northern bottlenose whales, Sowerby’s beaked whales (*Mesoplodon bidens*), Cuvier’s beaked whales, and a fourth click type resembling the clicks described for both True’s beaked whale (*M. mirus*) and Gervais’ beaked whale (*M. europaeus*), but not conclusively attributed to one of these species due to the similarities in signal characteristics^27^. We refer to this click type as ‘unidentified Mesoplodont beaked whale’ (UMBW) throughout this paper.

**Figure 2:**
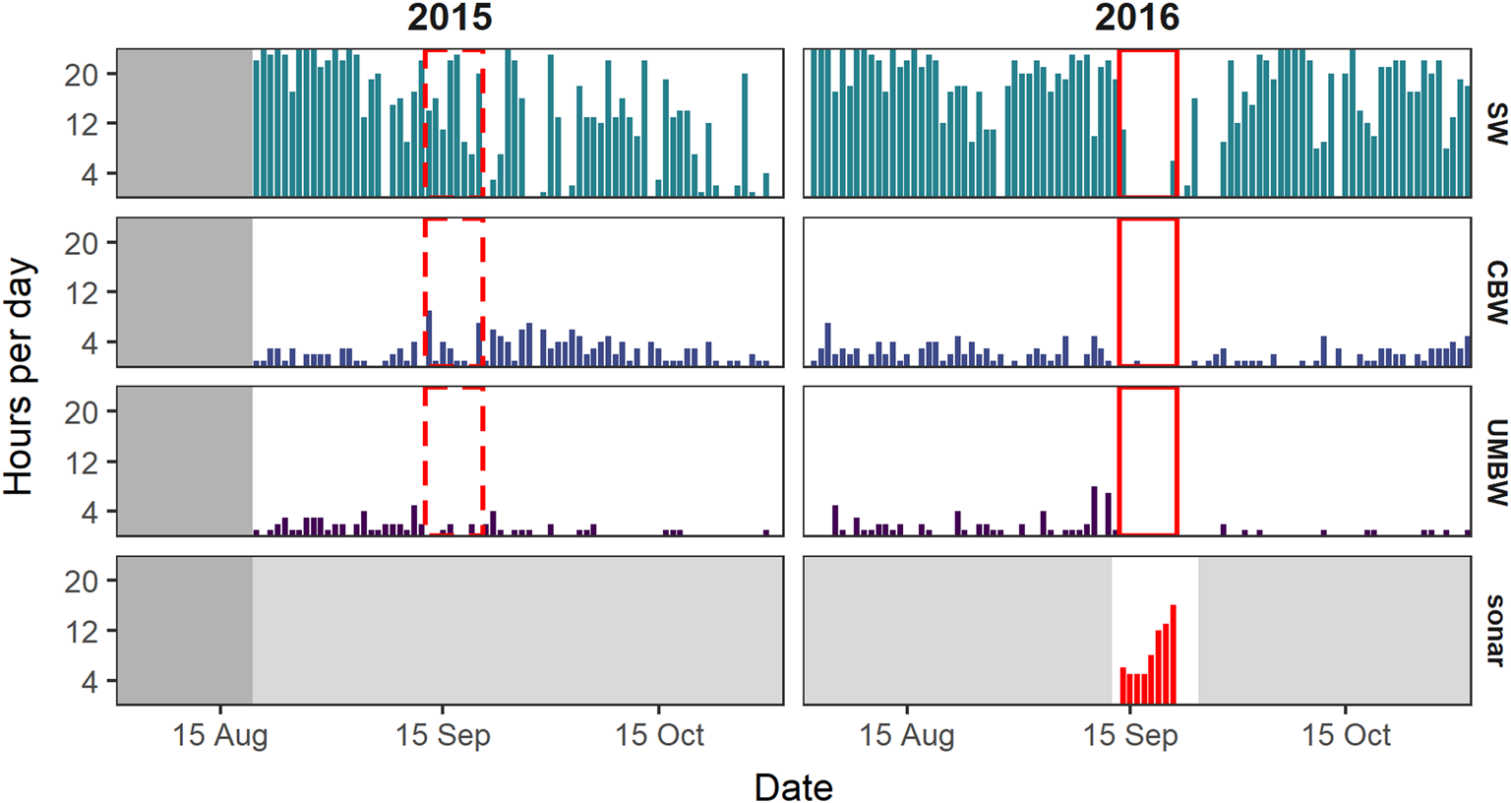
Number of hours per day with echolocation clicks from sperm whales (SW), Cuvier’s beaked whales (CBW), unidentified Mesoplodont beaked whales (UMBW), and sonar signals at the Station 5 recording site in 2015 and 2016. Dark grey shading in August 2015 indicates the period with no data available; light grey shading on the sonar plot indicates periods not analyzed for the presence of sonar signals. The solid red box delineates the period with sonar detections in 2016; the dashed box delineates the corresponding control period in 2015.

**Figure 3:**
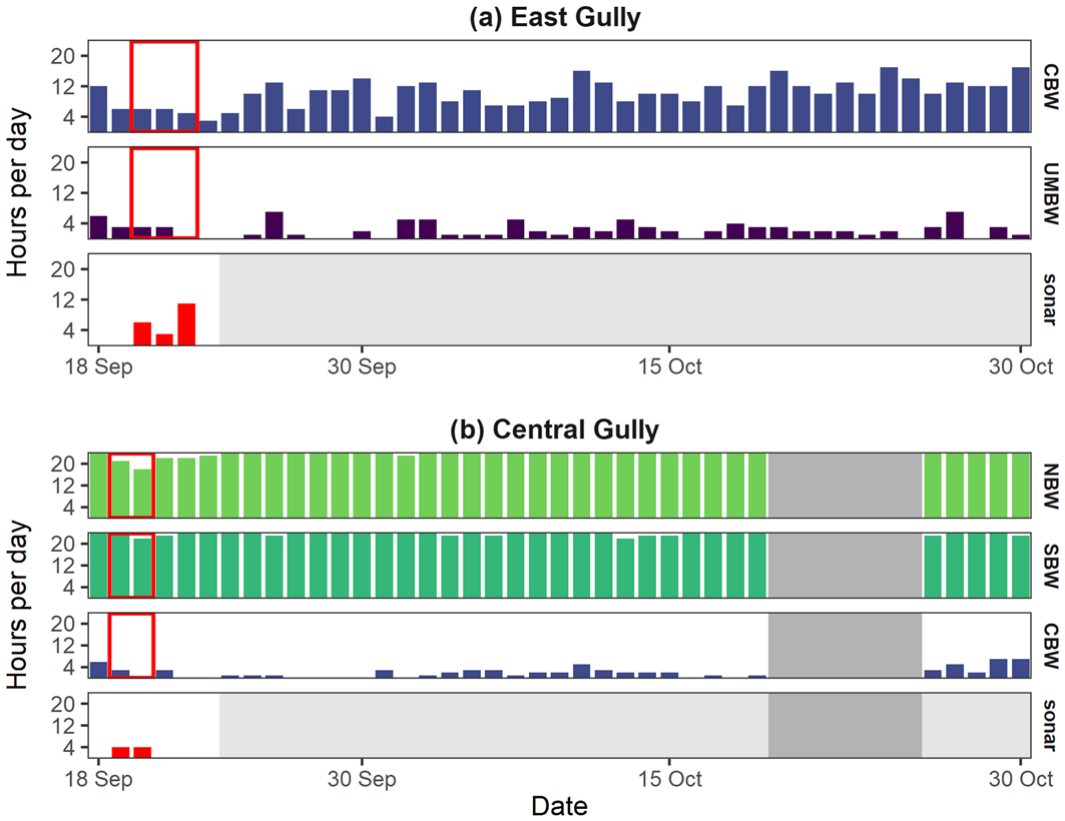
Number of hours per day with echolocation clicks from Cuvier’s beaked whales (CBW), unidentified Mesoplodont beaked whales (UMBW), northern bottlenose whales (NBW), Sowerby’s beaked whales (SBW), and sonar at the (**a**) East Gully and (**b**) Central Gully recording sites. Dark grey shading indicates periods of missing data; light grey shading on the sonar plots indicates periods not analyzed for the presence of sonar signals. The solid red box delineates the period with sonar signals present at each recording site.

The acoustic presence of species varied across recording sites. Sperm whale clicks were analyzed only at Station 5 and were commonly present at this site during the August to October analysis period in 2015 and 2016 (Table 1, Fig. 2). Cuvier’s beaked whale and UMBW clicks were more sporadic at Station 5, and present in fewer hours per day than sperm whales (Table 1, Fig. 2). Sowerby’s beaked whale clicks were detected once, on August 28th 2015, and there were no detections of northern bottlenose whale clicks at Station 5. At the East Gully site, Cuvier’s beaked whale clicks were the most prevalent beaked whale click type, present on all recording days in the September–October 2016 analysis period (Table 1, Fig. 3a). UMBW clicks were present less frequently, and clicks from Sowerby’s beaked whales and northern bottlenose whales were each detected once, on September 23rd 2016 and October 11th 2016, respectively. At the Central Gully site, patterns of beaked whale species occurrence were notably different from the other two locations, with clicks from northern bottlenose whales and Sowerby’s beaked whales present nearly 24 h per day throughout all available recording days (Table 1, Fig. 3b). There were relatively fewer hours per day with presence of Cuvier’s beaked whale clicks at this site; however, Cuvier’s beaked whale clicks may be slightly underrepresented in the results due to masking by high numbers of northern bottlenose whale clicks, which overlap in frequency. The East Gully and Central Gully recordings were of limited use for assessing changes in acoustic activity during the CF16 exercise, due to the lack of pre-exercise baseline data and the greater distance of these sites from the area of highest sonar activity. Because of these limitations and analysis time constraints, the East Gully and Central Gully recordings were not analyzed for the presence of sperm whale clicks.

**Table 1:** Summary of the number of days (percent of recording days) and mean number of hours per day (range: minimum–maximum) with echolocation clicks from Cuvier’s beaked whales (CBW), unknown Mesoplodont beaked whales (UMBW), Sowerby’s beaked whales (SBW), sperm whales (SW), and northern bottlenose whales (NBW) in each dataset analyzed.

Dataset	Species	Days (% recording days)	Mean h/day (range)
Station 5: Aug–Oct 2015 (*N* = 72 days)	CBW	61 (84.5%)	2.4 (0–9)
	UMBW	39 (54.2%)	1.0 (0–5)
	SBW	1 (1.4%)	0.01 (0–1)
	SW	63 (87.5%)	13.5 (0–24)
Station 5: Aug–Oct 2016 (*N* = 92 days)	CBW	65 (70.7%)	1.7 (0–7)
	UMBW	37 (40.2%)	0.8 (0–8)
	SW	80 (87.0%)	16.1 (0–24)
East Gully: Sep–Oct 2016 (*N* = 43 days)	CBW	43 (100%)	10.2 (3–17)
	UMBW	34 (79.1%)	2.3 (0–7)
	SBW	1 (2.3%)	0.02 (0–1)
	NBW	1 (2.3%)	0.02 (0–1)
Central Gully: Sep–Oct 2016 (*N* = 36 days)	CBW	26 (72.2%)	2.0 (0–7)
	SBW	36 (100%)	23.7 (22–24)
	NBW	36 (100%)	23.6 (18–24)

### Sonar signals and ambient noise levels

**Figure 4:**
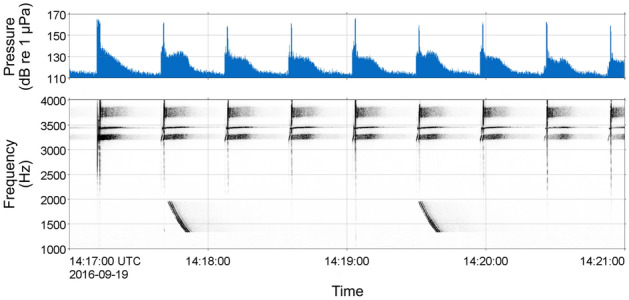
Timeseries (above) and spectrogram (below) displaying a sequence of sonar signals recorded over a 4-min period on September 19th 2016 at Station 5.

**Figure 5:**
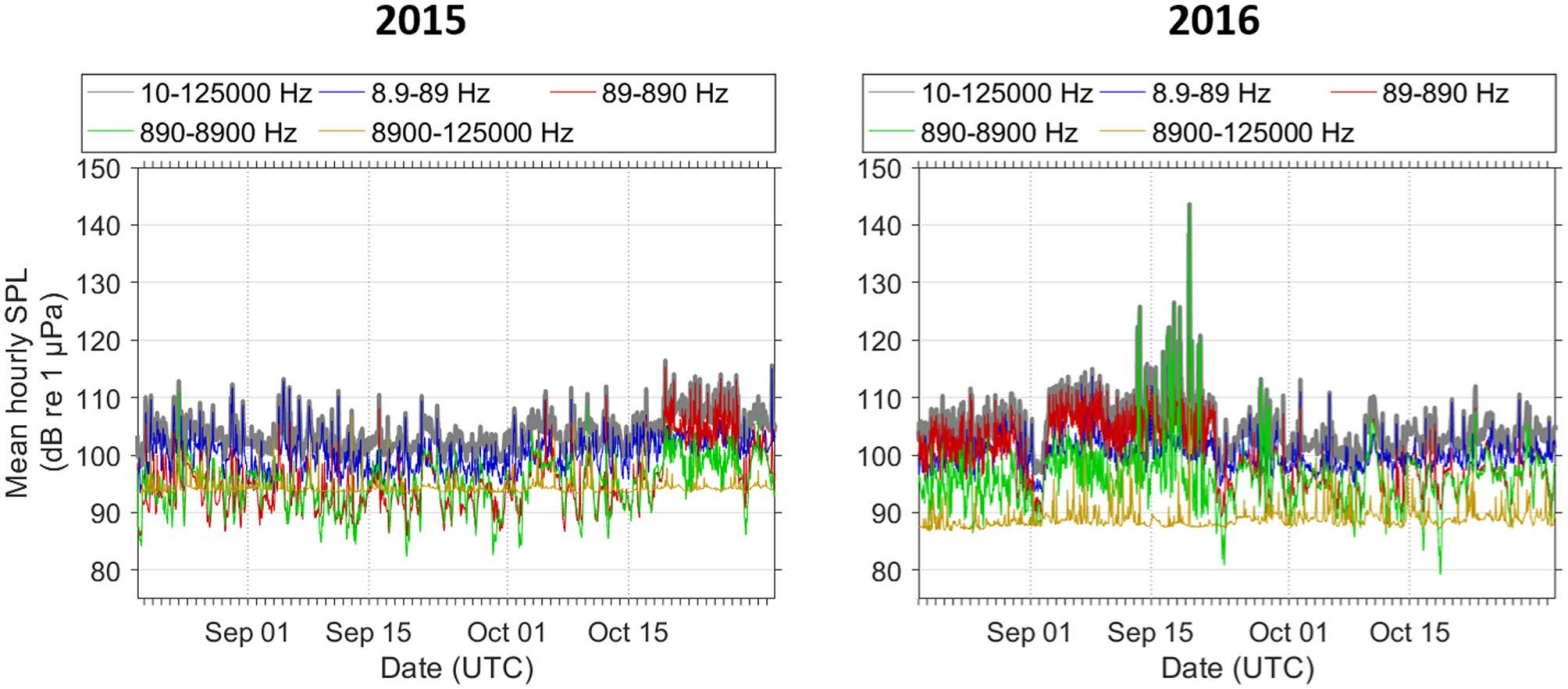
Mean hourly frequency band ambient sound pressure levels (SPL) recorded in 2015 and 2016 from August 19th to October 31st.

Naval active sonar signals were recorded at all three sites during the CF16 exercise period. Sonar activity was most prevalent at Station 5, where sonar signals were identified in a total of 70 h over an 8-day period from September 13th – 20th 2016. Sonar signals were recorded in 5 to 16 h per day during this period (Fig. 2), and generally occurred in bouts lasting from 1 to 13 h in duration. Table 2 provides a summary of measured attributes of the sonar signals recorded at each site. It is important to note the limited analysis frequency bandwidth at Station 5: approximately 94% of recordings at this site were made using an 8 kHz sampling rate and sonar signals extending above 4 kHz could not be fully measured in these recordings. A variety of signal types were recorded at Station 5, including frequency-modulated (FM) upsweeps and downsweeps, continuous-waveform signals, and more complex multi-part signals spanning multiple frequency bands (Fig. 4). Received sound pressure levels (SPL_200ms_) of annotated sonar signals were highest at Station 5, with a median SPL_200ms_ of 120 dB re 1 µPa (Table 2) and maximum SPL_200ms_ of 164 dB re 1 µPa. The prevalence of sonar signals at levels substantially above background noise was reflected in elevated mean hourly ambient noise levels in the 890–8900 Hz frequency band (Fig. 5). At the East Gully site, sonar signals were recorded in a total of 20 h over 3 days (September 20th –22nd 2016; Fig. 3a) and occurred in bouts lasting up to 13 consecutive hours. There was generally less variation in sonar signal attributes measured in the East Gully recordings, and received levels were lower than at Station 5, with a median SPL_200ms_ of 108 dB re 1 µPa (Table 2). The Central Gully recordings contained the fewest sonar signals, occurring in just one 4-h bout on September 19th and 4 additional hours on September 20th (Fig. 3b). The sonar signals recorded at Central Gully consisted of relatively faint FM downsweeps and upsweeps, with energy in a consistent frequency band between 1200 and 2000 Hz and lower measured received levels (median SPL_200ms_ 84 dB re 1 µPa; Table 2).

**Table 2:** Summary of measured attributes of sonar signals recorded at each site.

Attribute	Station 5	East Gully	Central Gully
Duration (s)	12.5 (2.5–21.4)	10.0 (4.9–14.4)	7.4 (5.2–15.5)
90% energy duration (s)	6.7 (1.0–14.5)	7.5 (3.4–11.3)	6.5 (4.6–14.1)
Low frequency (Hz)	2831 (1953–4890)	2420 (2272–3136)	1194 (1097–1303)
High frequency (Hz)	3873 (2800–7500)	3973 (3184–4482)	1974 (1765–2155)
Frequency bandwidth (Hz)	743 (357–1826)	1012 (716–2049)	742 (522–999)
Received SPL_200ms_ (dB re 1 µPa)	120 (92–138)	108 (86–119)	84 (82–87)
*N*	4622	1590	43

Median value and 10th–90th percentile range are provided for each attribute; *n* indicates the number of sonar signals annotated and measured at each site. Due to the use of a duty-cycled recording schedule, approximately 94% of the recordings collected at Station 5 had a limited analysis frequency bandwidth of 4 kHz.

### Changes in acoustic activity and ambient noise at Station 5 during the CF16 exercise

**Figure 6:**
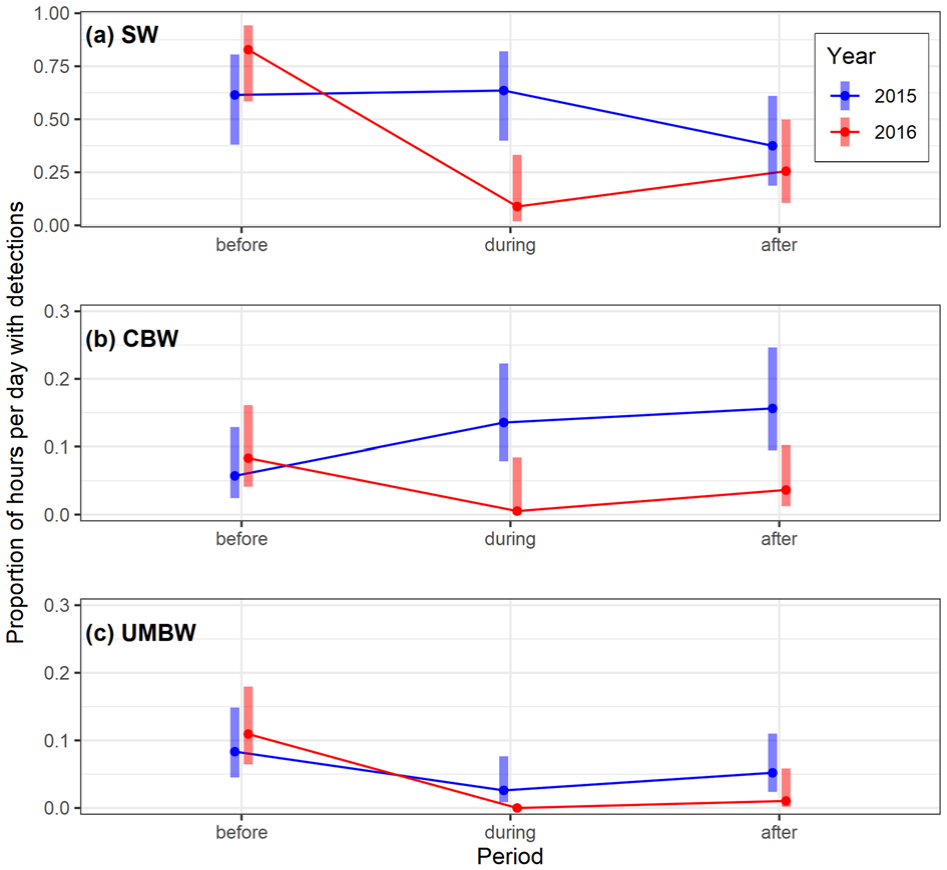
Interaction plots of the estimated marginal means and 95% confidence intervals from fitted GLM models testing the effects of period and year on the mean proportion of hours per day with echolocation clicks from (**a**) sperm whales (SW)*,* (**b**) Cuvier’s beaked whales (CBW), and (**c**) unidentified Mesoplodont beaked whales (UMBW) at the Station 5 recording site. Note that the y-axis scale differs across plots.

**Figure 7:**
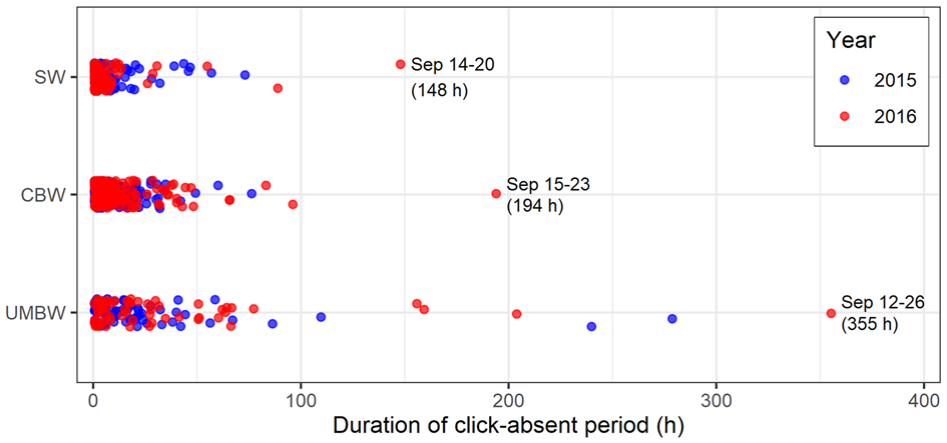
Distributions of click-absent period durations for unidentified Mesoplodont beaked whales (UMBW), Cuvier’s beaked whales (CBW) and sperm whales (SW) at Station 5 in 2015 (blue circles) and 2016 (red circles). Points are randomly jittered on y-axis for visibility. The most extreme outlier values for each species coincided with the CF16 exercise in September 2016 and are labeled with the date range and duration over which clicks were absent from the recordings.

Acoustic activity of sperm whales, Cuvier’s beaked whales, and UMBW differed across the 8-day periods before, during, and after the CF16 exercise in 2016 compared to control data from 2015, and there was a significant interaction between period and year in all models (Fig. 6; Supplementary Tables S1 & S2). Pairwise Tukey contrasts revealed a significant reduction in the acoustic activity of sperm whales recorded in the ‘during’ period in 2016 when compared to the ‘before’ period (*p* = 0.0005); sperm whale acoustic activity was also significantly lower in the ‘during’ period in 2016 than in the same period in 2015 (*p* = 0.0029). There were no significant differences between 2015 and 2016 in sperm whale acoustic activity recorded before or after the exercise period (Fig. 6). For the beaked whale species, lower baseline levels of acoustic activity made it more difficult to observe and quantify changes in the number of hours per day with clicks across periods. We did not find significant differences in the acoustic activity of Cuvier’s beaked whales across the ‘before’, ‘during’, and ‘after’ periods in 2016. However, the acoustic activity of Cuvier’s beaked whales differed between years, with significantly lower detection rates in the ‘during’ (*p* = 0.023) and ‘after’ (*p* = 0.012) periods in 2016 compared to the same periods in 2015. Due to the prevalence of zeros in the response variable for UMBW, it was not possible to estimate a 95% confidence interval for the mean response in the ‘during’ period in 2016 (Fig. 6) or perform pairwise Tukey contrasts across periods or years for this species. Notably, UMBW clicks were entirely absent from the recordings throughout the ‘during’ period and most of the ‘after’ period in 2016. Analysis of the duration of click-absent periods in the Station 5 recordings showed that UMBW clicks were absent from the recordings for a period of 355 h from September 12th to September 26th 2016, encompassing the full period with sonar signals from CF16 and an additional 7 days afterward. This period of absence represented a clear outlier, more than 75 h longer than the next longest click-absent period measured for this species (Fig. 7). Similarly, unusually long periods of absence for sperm whales (148 h) and Cuvier’s beaked whales (194 h) also coincided with the CF16 exercise, and in each case these represented maximal outlier values: 41 h and 98 h longer than the next longest click-absent period measured for each species, respectively (Fig. 7).

**Figure 8:**
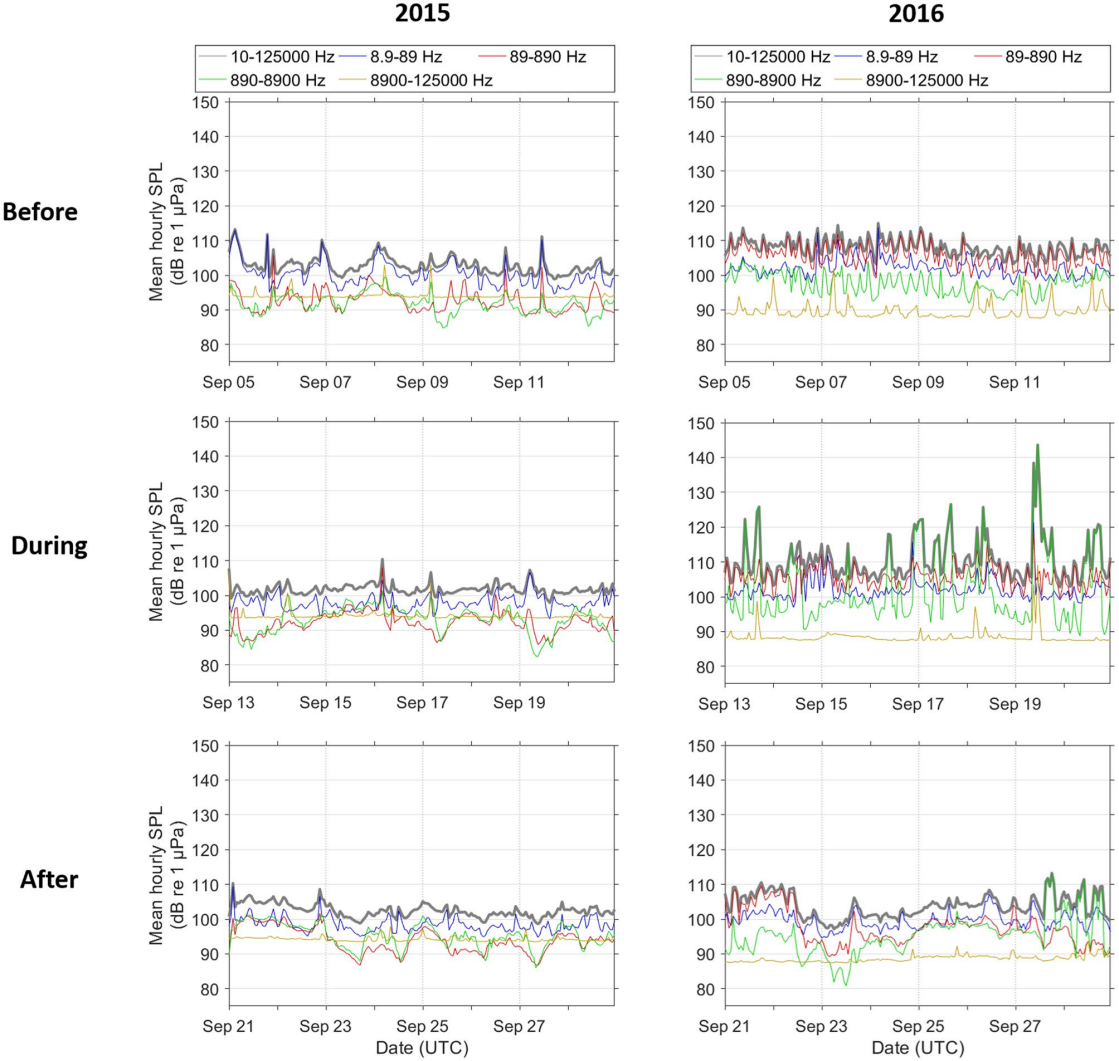
Mean hourly frequency band ambient sound pressure levels (SPL) recorded over the 8-day periods before (Sep 5th–Sep 12th), during (Sep 13th–Sep 20th), and after (Sep 21st–Sep 29th) the period in which sonar signals from the CF16 exercise were detected in 2016 (right) and the corresponding dates in 2015 (left).

A marked increase in mid-frequency ambient noise was measured in the ‘during’ period in 2016, reflecting the contribution of sonar signals to the soundscape during this time (Fig. 8). The 890–8900 Hz frequency band encompassed most of the energy from sonar signals, and was notably elevated throughout the CF16 exercise period.

## Discussion

We observed a clear reduction in the acoustic activity of sperm whales and beaked whales during the period when sonar signals were recorded at Station 5, indicating that whales ceased foraging in this area while military sonars were in use. The acoustic detection rate of sperm whales returned to pre-exercise baseline levels within the days following the CF16 exercise, while the observed reduction in beaked whale acoustic activity was more prolonged. Detection rates of Cuvier’s beaked whale clicks remained low throughout the 8-day period immediately following the exercise, and UMBW clicks were largely absent during this period. This study is observational and limited to showing correlation rather than cause and effect; nonetheless, these results are consistent with previous experimental research on the responses of beaked whales to simulated and real military sonars and suggest that whales were disturbed from normal foraging behaviour and likely displaced from the affected area during the CF16 exercise.

The scale and duration of sonar use recorded during this study provides important context for the observed results. Much of the experimental work conducted to date on the responses of beaked whales and other odontocetes to sonar has involved controlled exposure experiments using animal-borne tags to record the fine-scale movements and acoustic behavior of individuals, allowing responses to be examined on the scale of minutes to hours e.g.,^7,8,10^. Experimental exposures to simulated sonar signals lasting approximately 15–30 min have elicited pronounced avoidance responses in Blainville’s beaked whales^7^, Cuvier’s beaked whales^8^, Baird’s beaked whales^16^, and northern bottlenose whales^9,10^. Generally, these studies were focused on the onset of the response and did not always assess the duration over which altered behaviour continued. However, the absence of foraging behaviour for several hours following exposure was noted in some cases, and focal animals performed sustained directed movement away from the exposure location during this time, covering distances of up to tens of kilometers^10^. In broader-scale studies examining responses of Blainville’s beaked whales to real multi-ship naval training operations on the Atlantic Undersea Test and Evaluation Center (AUTEC) in the Bahamas, displacements of up to 68 km were observed, lasting 2–4 days before whales returned to foraging in the area where they were exposed^7,28^. In the present study, the duration of naval sonar activity recorded during the CF16 exercise was considerably more prolonged, with bouts of sonar continuing for up to 13 consecutive hours and occurring repeatedly over an 8-day period. Although we can only make inference on species-level rather than individual-level responses based on the absence of clicks in our recordings, it is plausible that military sonar activity at this scale led to wide spatial avoidance of the affected area over an extended period.

The absence of sperm whale click detections in the Station 5 recordings for 6 consecutive days during the CF16 exercise is notable; few prior studies have demonstrated sustained changes in foraging behaviour or substantial displacement of sperm whales following sonar exposure. Behavioural response studies conducted in northern Norway using controlled experimental exposures showed varying responses by sperm whales, which included changes in orientation and direction of horizontal movement, changes in acoustic behaviour, and altered dive profiles^23^. Exposure to lower frequency sonar signals in the 1–2 kHz range generally prompted stronger responses, including a reduction in foraging effort or transition from a foraging to non-foraging state, while exposure to higher frequency sonar signals in the range of 6–7 kHz did not appear to trigger changes in foraging behaviour^21,29^. More recently, Isojunno et al.^30^ quantified the responses of sperm whales to continuous and pulsed active sonars, and found that sound exposure level was more important than amplitude in predicting a change in foraging effort. We were not able to investigate differential responses to frequency or other sonar characteristics in this study, due to the observational nature of the study and the absence of sperm whale clicks throughout most of the exercise period. Likewise, we cannot exclude the physical presence of ships, aircraft, and submarines in the area or additional types of noise produced during maneuvers as potential factors contributing to the cessation of sperm whale and beaked whale click production and foraging behaviour.

The observed changes in acoustic activity were more easily quantified for sperm whales than for beaked whales, due to higher baseline hourly presence of sperm whale clicks in the recordings. Sperm whales produce powerful echolocation clicks throughout their foraging dives, which can be recorded at ranges of 16 km or more^31^, and a single individual foraging in the vicinity of a hydrophone may be detected continuously throughout multiple dive cycles. Our analysis was based on sperm whale click detections that met a threshold signal-to-noise ratio (SNR), and the results therefore provide a minimum estimate of sperm whale presence in the vicinity of the recorder. Reporting results at the level of hourly presence rather than the number of individual click detections largely mitigated the effects of excluding low-SNR clicks recorded at greater distances from the hydrophone or during higher ambient noise conditions. Likewise, the presence of sperm whales on an hourly time scale is not likely to be substantially underestimated when recordings are collected using a low duty cycle^32^. By contrast, beaked whales produce echolocation clicks at higher frequencies and lower source levels, with highly directional beam patterns^33^. These clicks are likely only detected at ranges of up to approximately 4 km when the whale is oriented toward the hydrophone, and at lesser distances when clicks are received off-axis^34^. As a result, there is greater variability and lower baseline detection rates of beaked whale clicks on fixed passive acoustic recorders, which reduces statistical power to assess temporal changes in acoustic activity. Moreover, the duty-cycled recording schedule used at Station 5 provided only 65 s of high-frequency data 3 times per hour, and the presence of beaked whales is likely to be underestimated by this duty cycle, with potentially greater underestimation of Mesoplodont species compared to Cuvier’s beaked whales^35^.

Continuous recordings were collected at the East Gully and Central Gully recording sites, but included only partial temporal coverage of the exercise period and no pre-exercise baseline data. No comparable recordings were available from these locations in a prior or subsequent year to form a control dataset. As a result, we were not able to use these datasets to assess changes in acoustic activity associated with the CF16 exercise. A slight decrease in hourly presence of northern bottlenose whale clicks in the Central Gully recordings occurred on September 19th–20th, 2016; however, we are aware that an oceanographic research vessel was coincidentally in the area deploying scientific instrumentation in close proximity to the Central Gully recording site on these dates, creating an additional source of potential disturbance. Despite these limitations, we included an analysis of the recordings collected at the East and Central Gully sites for two reasons: first, to provide perspective on the geographic extent over which activities associated with the CF16 exercise occurred; and second, to illustrate the diversity in beaked whale species composition at different locations across the region. Analysis of the recordings for sonar signals revealed that higher levels of sonar activity occurred near the Station 5 recording site than near the East or Central Gully locations. Due to the distance between recording sites and the timing of the sonar signals recorded, it appears that the recorded sonar signals came from multiple source locations over the duration of the exercise. Recordings from Central Gully contained the fewest sonar signals and lowest measured received levels, likely due to the deliberate avoidance of the Gully MPA and surrounding area by exercise participants during CF16. The Gully was established as an MPA in 2004, and is one of three adjacent canyons on the eastern Scotian Shelf currently designated as critical habitat areas for the endangered Scotian Shelf population of northern bottlenose whales^36^. The Station 5 recording site was located approximately 300 km to the southwest, and experienced higher levels of naval sonar activity during CF16. However, none of the locations were chosen specifically to monitor CF16, and we do not have access to information on the general exercise areas used, specific locations of naval vessels, submarines, or aircraft participating in the CF16 exercise, or the source levels of transmitted sonar signals. Due to the opportunistic nature of the recordings, the received levels of sonar signals measured at Station 5 likely do not represent the highest sound levels introduced into the marine environment during the CF16 exercise.

Unlike many areas where behavioural responses to sonar are commonly studied, there are no instrumented naval training ranges off eastern Canada, and cetaceans inhabiting this region are unlikely to be accustomed to regularly hearing naval active sonars. Other than during the CF16 exercise, sonar signals were not noted during a large-scale analysis of cetacean call occurrence and soundscape characterization in 2 years of recordings collected at Station 5 and numerous other passive acoustic monitoring sites off eastern Canada^26^. Exposure context and familiarity with a signal may be important factors influencing an individual’s response to acoustic disturbance^15^. Experimental research on Cuvier’s beaked whales near a U.S. naval training range located off southern California demonstrated possible distance-mediated effects of sonar exposure, with more pronounced behavioural responses occurring with closer source proximity, even when received levels from the closer source were likely lower than those from more distant, high-powered sonar transmissions, which did not elicit as strong a response^15^. The movement and predictability of the sound source as well as the timing and duration of sonar transmissions may also be important factors influencing the behavioural response^15^. Whales inhabiting waters off southern California are likely habituated to hearing distant sonar due to routine naval training activities occurring on the range. Conversely, Wensveen et al.^10^ found that northern bottlenose whales in the eastern North Atlantic exhibited similar responses to simulated sonar signals played at various distances up to 28 km, suggesting that they perceived this novel stimuli as a potential threat even from a distance and at relatively low received levels. Bernaldo de Quiros et al.^5^ hypothesized that beaked whales not regularly exposed to active sonar signals may respond more strongly, both physiologically and behaviourally, which poses a concern for a region where military training activities involving the use of sonar are relatively infrequent, but occur periodically in the form of large-scale exercises involving the extensive use of active sonars and creating significant potential for acoustic disturbance.

Behavioural disturbance due to anthropogenic noise may have energetic, health, and fitness consequences for deep-diving odontocete species. Disruption of normal diving patterns creates energetic costs due to the significant investment in each dive and the reduction of time available for prey intake when foraging dives are interrupted. Recent studies on the functional relationship between beaked whales and deep-sea prey resources suggest that certain characteristics of prey, including minimum size and density thresholds, are required for beaked whales to successfully meet their energetic needs^12,37^. While the distribution and characteristics of deep-sea prey are challenging to study and largely unknown in most regions, considerable environmental heterogeneity may be present, causing the quality of foraging habitat to vary significantly over even small horizontal scales^12,37^. This patchiness in habitat quality has important implications for behavioural disturbance, as even short-term displacement from high-quality habitat areas can affect the fitness of individuals and potentially lead to population-level consequences^13^.

In addition to the consequences of sublethal disturbance, it is important to note that the likelihood of observing more acute impacts of exposure to naval active sonar, including injuries or fatalities, is extremely low in offshore regions. Individual and mass strandings of beaked whales and other cetaceans associated with military activities have typically been documented on oceanic islands with populated coastlines^1,3,6^. Factors affecting the probability that cetacean carcasses will wash ashore include buoyancy and decomposition rates in local water conditions, oceanic surface currents, the topography of coastlines, and the location of habitat relative to shore^6^. Off Nova Scotia, potential beaked whale and sperm whale habitat (consisting of water depths greater than 500 m) is located more than 100 km from the coastline, and injuries or fatalities occurring in deep water habitat in this region are unlikely to result in observed strandings. Stranding incidents involving sperm whales and beaked whales have been reported in Nova Scotia, but the cause of mortality is usually unknown^38^. Cetacean mortality is highly underestimated even in the aftermath of catastrophic events such as large oil spills^39^, and a lack of observed injuries or mortalities following offshore military activities should not be construed as evidence that no direct or immediate harm was caused.

This study offered a unique opportunity to use existing passive acoustic monitoring (PAM) data to assess disturbance of poorly-known odontocete species during a real-world, large-scale military sonar exercise in a region where military sonar use at this scale is relatively uncommon. Ideally, a PAM study designed to examine disturbance in this context would collect continuous rather than duty-cycled recordings, and include ample baseline data surrounding the period of interest as well as in prior and subsequent years. Additionally, multiple acoustic sensors arranged in a dense array surrounding exercise locations would provide further insight into the spatial context of exposure and patterns of disturbance. Despite the data limitations in the present study, our results demonstrate that changes in odontocete foraging behaviour associated with acute, large-scale disturbance may be evident in PAM data even at low duty cycles. The nature of the observed effect (e.g., temporary disruption of foraging, spatial displacement, or more acute injury or distress) remains unknown, as do the number of individuals affected and the longer-term health and fitness implications. Broader baseline data on species occurrence and an improved understanding of species’ ecology and habitat use in the region are necessary for making informed mitigation decisions, allowing key habitat areas to be avoided, and understanding the impacts of naval active sonar exposure in this region on individuals and populations.

## Methods

### Acoustic data collection

Data for this study consisted of passive acoustic recordings collected with archival bottom-mounted Autonomous Multichannel Acoustic Recorders (AMARs) (JASCO Applied Sciences, Inc.) deployed at three locations along the continental slope off the Scotian Shelf in eastern Canada (Fig. 1). The primary dataset comprised recordings collected at Station 5, located at 42.55 N, 62.18 W at a depth of approximately 1800 m. Data were collected at this site as part of a broad-scale passive acoustic monitoring project occurring off eastern Canada from August 2015 to July 2017^26^. The AMAR deployed at Station 5 was fitted with an HTI-99 omnidirectional hydrophone (High Tech, Inc.; nominal sensitivity level of -162 ± 3 dB re 1 V/μPa) in 2015, and a GTI M36-V35-100 omnidirectional hydrophone (GeoSpectrum Technologies, Inc.; nominal sensitivity level of -165 ± 3 dB re 1 V/μPa) in 2016. Recordings were made using a duty-cycled recording schedule, sampling at 8 kHz for 11 min 18 s, then at 250 kHz for 1 min 4 s, repeating every 20 min. For this study, we analyzed recordings collected from August through October in both 2015 and 2016, with 2015 treated as a control year prior to the CF16 exercise occurring in September 2016.

Additional recordings were collected at two sites off the eastern Scotian Shelf: Central Gully (43.86 N, 58.89 W; 1500 m depth), located inside the Gully MPA, and East Gully (43.87 N, 58.00 W; 2000 m depth), located 72 km to the east (Fig. 1). At each of these sites, an AMAR was fitted with a GTI M36-V35-100 hydrophone, and recordings were made continuously from September 18th 2016 to October 30th 2016 at a sampling rate of 250 kHz. Due to an instrument malfunction, no data were recorded at the Central Gully site from October 19th –25th 2016.

### Detection and classification of beaked whale and sperm whale clicks

To detect and identify beaked whale echolocation clicks, we used a multi-step process involving automated click detection, followed by manual review, classification of the detected clicks to the species level, and verification of species presence per hour. This process was based largely on the methods previously described by Baumann-Pickering et al.^40,41^, and Stanistreet et al.^25^ using modified click discrimination criteria to improve the detection and identification of target beaked whale species in the western North Atlantic region. The first step was to apply an automated algorithm to all high-frequency recordings (250 kHz sampling rate) to detect echolocation clicks^42^. For the Station 5 and East Gully recordings, where beaked whale presence was relatively sporadic, we subsequently applied a set of ‘general’ beaked whale click discrimination criteria to identify potential beaked whale clicks based on measured attributes (see Supplementary Table S3), and used summary figures and spectrograms of clicks to manually verify and classify them to species. For the Central Gully recordings, which were dominated by high numbers of northern bottlenose whale clicks that tended to mask other click types, we applied species-specific criteria to facilitate manual review of clicks likely produced by northern bottlenose whales, Sowerby’s beaked whales, and Cuvier’s beaked whales (see Supplementary Table S3). These criteria were not intended to automatically classify all beaked whale clicks to the species level, but rather allowed us to perform an efficient manual review by initially examining the most likely candidate clicks for each species. In all cases, potential beaked whale click detections were binned by calendar hour (for duty-cycled recordings, each hour consisted of 3 sampling periods). Detections within each hour were manually reviewed and classified to the species level to verify the hourly presence of each species. Hours were marked with species absence if there were no automated detections of potential beaked whale clicks or if all detections were determined to be false during manual review. For each species, the number of hours per day with verified click detections was used for data visualization and statistical analysis.

To detect sperm whale clicks in the high-frequency recordings collected at Station 5, we used a signal-to-noise-ratio (SNR)-based automated detection algorithm, described in detail by Beslin et al.^43^. For each detection, the click waveform and a noise sample were extracted from the audio files and filtered using a 2–120 kHz Butterworth band pass filter. We applied a set of frequency-based criteria to identify potential sperm whale clicks from the candidate detections (see Supplementary Table S3). Sperm whale detection events were created for each audio segment containing clicks which met these criteria and had a median inter-click-interval of 2 s or less. These detection events were binned by hour and manually reviewed to confirm the presence of sperm whale clicks on an hourly basis. Initially, we performed this manual review using summary figures of click attributes and spectrograms of individual clicks, as was done for the beaked whale analysis. However, we found that it was often necessary to review spectrograms in the context of the original audio file and listen to click sequences to confidently verify sperm whale presence, particularly when other sources of noise were present. False detections of sperm whale clicks triggered by sonar signals during CF16 and higher ambient noise levels present during the exercise prompted extra manual review of the recordings collected during the exercise period to verify the hourly presence or absence of sperm whale clicks. Sperm whale clicks are visible on a spectrogram at lower SNRs than the threshold required for automated detection, and manual review of spectrograms allowed us to confirm that sperm whale click presence was not underestimated during the exercise period due to reduced SNR or masking of clicks by sonar signals. Outside of the exercise period, all hours with sperm whale detection events were manually reviewed to verify presence, and hours with no detections were marked with species absence. The number of hours per day containing verified sperm whale click detections was used for data visualization and statistical analysis.

### Analysis of mid-frequency active sonar signals and ambient noise

To identify and characterize naval active sonar signals in the recordings, acoustic data collected at both the 8 kHz and 250 kHz sampling rates from September 12th to 23rd 2016 were manually reviewed using PAMlab software (JASCO Applied Sciences Inc., Dartmouth, Nova Scotia). Acoustic files were viewed as spectrograms using a 2 Hz frequency resolution, 0.128 s time window, 0.32 s time step, and a Hamming window. Sonar signals were manually annotated on the spectrogram using a box encompassing the entire visible signal. Due to the duty-cycled recording schedule, approximately 94% of the recordings from Station 5 were made using a sampling rate of 8 kHz, limiting the analysis frequency bandwidth to 4 kHz. The remaining recordings were made with a sampling rate of 250 kHz. For each identified sonar signal, the following attributes were measured: duration of annotated signal, duration containing 90% of the energy within the annotation box, minimum frequency, maximum frequency, frequency bandwidth, and received sound pressure level (SPL; dB re 1 µPa). To measure sonar SPL, we aimed for consistency with previous studies and followed the methods summarized by Von-Benda Beckmann et al.^44^: RMS signal level was calculated using a 200 ms sliding window stepping in 20 ms increments over the sonar signal duration, and the maximum value of this 200 ms integration window was reported as SPL_200ms_ for the annotated sonar signal.

To measure ambient noise in the Station 5 recordings, sound pressure levels were computed for each 60 s of acoustic data^45^. This was accomplished by computing 120 fast-Fourier transforms (1 s of data; 50% overlap; Hann window; 1 Hz resolution) that were subsequently averaged and stored as mean 60 s power spectral densities. The power spectral density data were summed to produce 60 s frequency bands with the following bandwidths: 8.9–89 Hz, 89–890 Hz, 890–8,900 Hz and 8,900–125,000 Hz as well as total broadband SPL (10–125,000 Hz). Hourly mean ambient sound pressure levels were then calculated for each frequency band.

### Assessing changes in acoustic activity during the CF16 exercise

Using the Station 5 dataset, we conducted a statistical analysis to compare the acoustic activity of each species before, during, and after the occurrence of sonar signals recorded in September 2016. The ‘before’ period was defined as September 5th-12th, the ‘during’ period as September 13th-20th, corresponding to the dates in which sonar signals were present in the recordings, and the ‘after’ period as September 21st-28th. Recordings from corresponding dates in 2015 provided a control dataset for this comparison. We used quasi-binomial generalized linear models (GLMs) to examine the effects of the interaction between period (categorical with 3 levels: ‘before’, ‘during’, ‘after’) and year (categorical with 2 levels: 2015 or 2016) on the response variable: proportion of hours per day with species presence. Separate models were fit for each species. We used the ‘acf’ function in R^46^ to check the GLM model assumption of independence, and found no evidence of temporal autocorrelation in model residuals. To test for differences in acoustic activity across periods and years, we used the ‘emmeans’ package in R^47^ to estimate marginal means and 95% confidence intervals from fitted GLM models and perform pairwise Tukey contrasts. Ambient noise plots were produced for the same time periods to illustrate the differences in noise levels across periods in 2015 and 2016 and the contribution of naval active sonar activity.

The cessation of echolocation is an anticipated response to disturbance, and the periods of time over which verified click detections were absent from the recordings were also of interest. Using the Station 5 recordings from August through October in both 2015 and 2016, we measured the duration of all click-absent periods for each species by summing the number of consecutive ‘absence’ hours occurring between each subsequent ‘presence’ hour for a given species. The distributions of click-absent period durations for each species in each year were plotted to visualize and identify outlier values representing unusually long periods of absence.

## Supplementary Information

Supplementary Information.

## Data Availability

The datasets generated during and/or analysed during this study can be made available upon reasonable request.
