# Supplementary material for: Changes in the acoustic activity of beaked whales and sperm whales recorded during a naval training exercise off eastern Canada
Source: Sci Rep. 2022 Feb 7;12:1973. doi: 10.1038/s41598-022-05930-4 (PMC8821608; doi:10.1038/s41598-022-05930-4)
Supplement: Supplementary file 1 — Supplementary Information. [file 41598_2022_5930_MOESM1_ESM.pdf]

Changes in the acoustic activity of beaked whales and sperm whales recorded during a naval training exercise off eastern Canada

Joy E. Stanistreet[1*], Wilfried A. M. Beslin[1], Katie Kowarski[2], S. Bruce Martin[2], Annabel Westell[1], and Hilary B. Moors-Murphy[1]

[1] Fisheries and Oceans Canada, Bedford Institute of Oceanography, 1 Challenger Drive, Dartmouth, Nova Scotia, Canada

[2] JASCO Applied Sciences, 32 Troop Avenue, Suite 202, Dartmouth, Nova Scotia, Canada

*Corresponding author: joy.stanistreet@dfo-mpo.gc.ca

Supplementary Information

Table S1. Analysis of deviance tables using Type III tests for GLMs testing the interaction between 'year' and 'period' on the proportion of hours per day with echolocation clicks from sperm whales, Cuvier's beaked whales, and unidentified Mesoplodont beaked whales.

	**Likelihood Ratio chi sq**	**df**	***p***
Sperm whales			
Period	3.0203	2	0.22
Year	2.0603	1	0.15
Period*Year	12.4198	2	0.0020
Cuvier's beaked whales			
Period	5.2459	2	0.073
Year	0.4688	1	0.49
Period*Year	11.9921	2	0.0025
Unidentified Mesoplodont beaked whales			
Period	3.9459	2	0.14
Year	0.4616	1	0.50
Period*Year	7.6683	2	0.022

Table S2. Results of pairwise contrasts using estimated marginal means from the GLM models for sperm whales and Cuvier's beaked whales. Results are shown on the log odds ratio scale. P-values were adjusted by the Tukey method for comparing a family of three estimates.

Contrast	Odds ratio	SE	*z* ratio	*p*
Sperm whales: test period by year				
2015: before / during	0.915	0.633	-0.128	0.99
2015: before / after	2.658	1.835	1.416	0.33
2015: during / after	2.905	2.016	1.536	0.27
2016: before / during	49.599	51.785	3.739	0.0005
2016: before / after	14.061	11.679	3.183	0.0042
2016: during / after	0.283	0.282	-1.266	0.41
Sperm whales: year by test period				
Before: 2015 / 2016	0.331	0.263	-1.392	0.16
During: 2015 / 2016	17.941	17.376	2.981	0.0029
After: 2015 / 2016	1.751	1.281	0.766	0.44
Cuvier's beaked whales: test period by year				
2015: before / during	0.388	0.213	-1.726	0.20
2015: before / after	0.328	0.177	-2.068	0.097
2015: during / after	0.846	0.358	-0.395	0.92
2016: before / during	17.364	26.283	1.886	0.14
2016: before / after	2.403	1.634	1.289	0.40
2016: during / after	0.138	0.217	-1.260	0.42
Cuvier's beaked whales: year by test period				
Before: 2015 / 2016	0.669	0.396	-0.679	0.50
During: 2015 / 2016	29.916	44.779	2.270	0.023
After: 2015 / 2016	4.894	3.099	2.508	0.012

Table S3. Criteria used to identify potential beaked and sperm whale whale clicks prior to manual verification. Single values represent minimum thresholds applied with no upper limit; ranges of values indicate minimum and maximum limits. References which further describe the calculation of attributes are provided in the footnotes.

Attribute	Beaked whale: general	Northern bottlenose whale	Sowerby's beaked whale	Cuvier's beaked whale	Sperm whale
Peak frequency (kHz)[1]	> 23	23 - 36	> 55	30 - 46	< 20
Center frequency (kHz)[1]	> 23	23 - 36	> 55	31 - 42	< 20
-10 dB bandwidth (kHz)[1]	> 10	10 - 35	10 - 40	10 - 30	< 25
-10 dB lower freq. bound (kHz)[2]	> 15	15 - 25	> 45	17 - 35	< 15
-10 dB upper freq. bound (kHz)[2]	> 35	30 - 45	> 70	40 - 55	< 28
Zero-crossing rate (ms^{-1})	> 50	40 - 80	> 90	50 - 85	*NA*
Duration (ms)[3]	> 0.25	> 0.3	0.20 - 0.60	> 0.25	*NA*
Sweep rate (kHz/ms)[4]	> 25	35 - 100	> 25	> 25	*NA*
Sweep rate duration (ms)[4]	> 0.16	> 0.18	0.16 - 0.24	> 0.16	*NA*
50% energy duration (ms)[5]	> 0.085	0.10 - 0.35	0.05 - 0.35	0.08 - 0.20	*NA*

[1] Baumann-Pickering, S. *et al.* Species-specific beaked whale echolocation signals. *J. Acoust. Soc. Am.* **134**, 2293–2301 (2013).

[2] DeAngelis, A. I., Stanistreet, J. E., Baumann-Pickering, S. & Cholewiak, D. M. A description of echolocation clicks recorded in the presence of True's beaked whale (Mesoplodon mirus). *J. Acoust. Soc. Am.* **144**, 2691–2700 (2018).

[3] Soldevilla, M. S. *et al.* Classification of Risso's and Pacific white-sided dolphins using spectral properties of echolocation clicks. *J. Acoust. Soc. Am.* **124**, 609–624 (2008).

[4] Baumann-Pickering, S., Trickey, J. S., Wiggins, S. M. & Oleson, E. M. Odontocete occurrence in relation to changes in oceanography at a remote equatorial Pacific seamount. *Mar. Mammal Sci.* **32**, 805–825 (2016).

[5] Stanistreet, J. E. *et al.* Using passive acoustic monitoring to document the distribution of beaked whale species in the western North Atlantic Ocean. *Can. J. Fish. Aquat. Sci.* **74**, 2098–2109 (2017).
